# Covalent Inhibitors in Antimicrobial Drug Development—Beyond β-Lactams

**DOI:** 10.3390/molecules31122186

**Published:** 2026-06-22

**Authors:** Ghazaleh Jafari, Dustin Duncan

**Affiliations:** Department of Chemistry, Brock University, St. Catharines, ON L2S 3A1, Canada; vw25ae@brocku.ca

**Keywords:** antibacterial, antifungal, antiparasitic, antiviral, chemical biology, covalent inhibitor, drug development, drug discovery

## Abstract

For nearly a century, since the discovery of penicillin by Alexander Fleming, we have used covalent inhibitors as antimicrobial drugs. The success of penicillin in treating microbial infections led to numerous other antibiotics containing β-lactam, the reactive warhead that forms the covalent adduct, exemplified by later-generation cephalosporins with approvals into 2020. In parallel, early non-β-lactam covalent agents also emerged, extending covalent mechanisms beyond β-lactam antibacterials to antifungal, antiparasitic, and antiviral applications. Despite the successes of covalent mechanisms of action, there are still considerable safety concerns due to the possibility of off-target covalent adducts which may lead to significant side effects. This review provides an overview of non-β-lactam covalent antimicrobials across all major pathogen classes, organized by their warhead class, covalency, and resistance mechanisms, and outlines design and clinical-level mitigation strategies. We trace the field from the serendipitous discovery of penicillin to the intentional design of new drugs, with a discussion of changes in perception and evolution of technology that enable modern covalent drug design.

## 1. Introduction

It has been approximately a century since the serendipitous discovery of penicillin. This discovery served as a turning point for combating microbial infection and initiated the Golden Age of antibiotics discovery [1,2]. Penicillin and other β-lactam antibiotics have a covalent mode of action wherein a serine residue of penicillin-binding proteins performs a ring-opening reaction on the highly strained four-membered lactam ring [3]. This mechanism of action has been widely replicated in developing “next-generation” penicillin, cephalosporins, carbapenems, and monobactams to broaden the spectrum of bacteria to target and to overcome resistance mechanisms that the bacteria have evolved [4,5].

β-lactam antibiotics have been reviewed extensively from numerous perspectives [4,6,7]. To date, in clinical practice, the covalent inhibition paradigm has been mainly dedicated to β-lactam scaffolds, and only a few non-β-lactam antimicrobial drugs have achieved clinical approval such as nitrofurans [8], cycloserine [9], isoniazid [10], and fosfomycin [11] among antibacterials; tavaborole [12] among antifungals; eflornithine [13], and nitroimidazoles [14,15] such as metronidazole among antiparasitics; and boceprevir [16], telaprevir [17], and nirmatrelvir [18,19] among antivirals. Since the extension of covalent inhibition strategies to non-β-lactam scaffolds is not yet sufficiently developed, it has led to a significant gap in drug discovery [20,21].

Covalent inhibitors use an electrophilic warhead to form a covalent bond with a biological target, commonly a nucleophilic residue in or near an enzyme active site [22,23,24]. This covalent interaction may be reversible or irreversible depending on the rate of the reverse reaction [20]. In targeted covalent inhibition, potency depends on both the reversible recognition step and the subsequent bond-forming step: *k*_1_ and *k*_−1_ describe inhibitor binding and dissociation, *K_I_* describes the affinity of the reversible inhibitor–target complex, and *k_inact_* describes the rate of covalent inactivation by the warhead [25].

In antimicrobial drug discovery, when the warhead is appropriately positioned, covalent inhibition can increase apparent potency and prolong target engagement. However, uncontrolled reactivity can cause nonspecific or off-target modification, including nonspecific thiol trapping and reaction with glutathione [26]; therefore, rational design requires balancing electrophilicity, binding-site recognition, and exposure [22,23,24].

Here, we review the development of non-β-lactam covalent antimicrobial drugs and the significant changes in scientific attitudes from the 20th century to present-day design. Advances in technologies (e.g., structural biology, high-throughput screening, computational modelling, and artificial intelligence) have led to a renaissance in covalency as a desired drug mechanism and opened opportunities for the rational design of novel non-β-lactam covalent inhibitors in the antibacterial, antifungal, antiviral, and antiparasitic applications. These factors have been organized based on their warheads, covalency (reversible/irreversible), resistance mechanisms, design and clinical mitigation strategies, and future directions.

## 2. Development of Covalent Antimicrobials Throughout History

### 2.1. 1920s–1950s (Serendipitous Discoveries)

Drug discovery and development during this period was not performed through rational design but rather through phenotypic screens of natural products. This approach was reasonably rapid since it allowed for determining which extracts are bioactive and then isolating the active compounds. This experimental strategy marked the early “golden age” of antibiotics [2].

**Figure 1:**
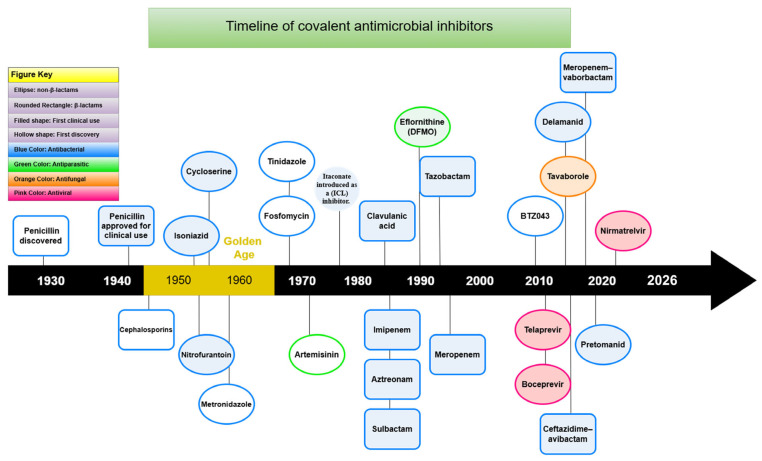
Timeline of covalent antimicrobial inhibitors. Itaconate (1977) is shown as a host-derived mechanistic milestone rather than a drug.

**Figure 2:**
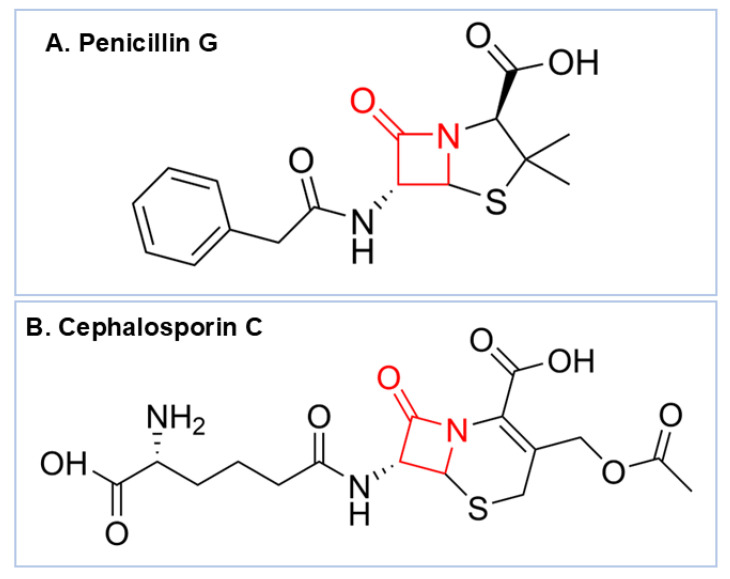
Representative β-lactam covalent antimicrobial agents from 1920s–1950s. β-lactam warheads are highlighted in red.

The discovery of penicillin in 1928 was a remarkable success (Figure 1): Once developed into a systemic treatment, it became one of the most important weapons ever used to combat infectious diseases and helped establish systematic screening of microbial extracts as an effective strategy for discovering additional antibiotics [27,28]. The screening of microbial extracts led to the establishment of numerous new classes of antibiotics, including aminoglycosides (streptomycin, 1943) [29], chloramphenicol (Chloromycetin,1947) [30], tetracyclines (Aureomycin, 1948) [31], macrolides (Ilotycin/erythromycin, 1952) [32], streptogramins (pristinamycin, 1953) [33], rifamycins (1957) [34], and glycopeptides (vancomycin, 1958) [35]. During this period, antifungal polyenes such as nystatin (1950) [36] and amphotericin B (1955) [37] were also discovered. Most of the classifications in this era belong to non-covalent inhibitors; however, a few notable covalent agents were also found. Natural-product β-lactams such as Penicillin G [3,38] and cephalosporin C (Figure 2) [39] were later found to act through covalent acylation of penicillin-binding proteins, while D-cycloserine [9,40] acts through pyridoxal 5′-phosphate (PLP)-trapping of cell-wall enzymes. Also, isoniazid [10] and nitrofurantoin [8] represent some of the early synthetically derived covalent agents. Although covalent chemistry in drug design existed in this period, it was mostly discovered serendipitously rather than through rational design [20].

During this time, the chemical characterization of the bioactive compounds was not typically associated with structural elucidation, given the available techniques at the time. Nuclear magnetic resonance (NMR) was not yet widely and conventionally available for structural elucidation, and its widespread use occurred in the 1950s [41]. State-of-the-Art chemical elucidation was primarily determined through chemical decomposition of products into known and characterized compounds, in addition to X-ray crystallography for bioactive natural products that could crystallize [42]. Furthermore, the field of organic synthesis was relatively undeveloped. For example, the total synthesis of strychnine, a complex natural product, was not achieved until 1954 by R.B. Woodward [43], and retrosynthetic analysis was not formalized until 1967 [44]. Similarly, cell and systems biology had not made significant progress. Even enzyme kinetics was still in its infancy. For example, the Lineweaver–Burk diagram was introduced in 1934 [45]. The intentional design of covalent inhibitors was impractical due to the lack of characterization of protein structures [46,47]. Therefore, these scientific limitations and the deficit of chemical synthesis tools led to the exploration of covalent inhibition remaining largely haphazard, relying on natural products with occasional covalent mechanisms of action [2,20].

### 2.2. 1960s–1970s (The Rise in Rational Design and Risk Aversion)

At this time, organic synthesis advanced significantly: the formalization of retrosynthetic analysis provided a systematic framework for planning complex molecule construction [44]. Additionally, advancements in NMR spectroscopy greatly accelerated the characterization process [41].

In parallel, the U.S. Food and Drug Administration (FDA) enhanced its requirements for evidence of drug safety and efficacy, especially in light of the use of thalidomide in the early 1960s for treating nausea [48]. This risk aversion poured cold water onto the field of covalent drug discovery due to intrinsic reactivity and significant possibility of off-target effects [20]. In principle, these off-target effects can be circumvented through effective rational design with the concept of the pharmacophore. Although introduced in the early 1900s by Paul Ehrlich [49], a modern definition was proposed by Gund in 1977 as a set of structural features in a ligand that are recognized at the receptor site and are responsible for its biological activity [50]. Regarding that, pharmacophore modelling by the mid-1970s became a ligand-based practical tool through encoding the spatial arrangement of noncovalent features required for recognition, providing 3D alignment and the pre-screening of large libraries even without a protein structure [51]. Together, 3D-pharmacophore methods and active-analogue alignment provided the first computational frameworks for ligand-based discovery [52,53].

A clear example of rational covalent inhibitor design from this period is eflornithine (α-difluoromethylornithine, DFMO; 1978). DFMO was reported as a substrate/product analogue and catalytic irreversible inhibitor of ornithine decarboxylase [54].

Several other covalent antimicrobial mechanisms were discovered or introduced during this period, including metronidazole (first reported in 1959 and first clinical use in the 1960s) [14], fosfomycin (1969) [11], and tinidazole (1969) [55] among antibacterials, and artemisinin (1972) [56] among antiparasitic. Itaconate was later reported as an inhibitor of bacterial isocitrate lyase (ICL), then in 2011 it was discovered to be a macrophage-derived metabolite [57]. Recent work has shown that itaconate acts as a covalent inhibitor of *Mycobacterium tuberculosis* Icl1 by an active-site cysteine adduct formation (Figure 1) [58].

**Figure 3:**
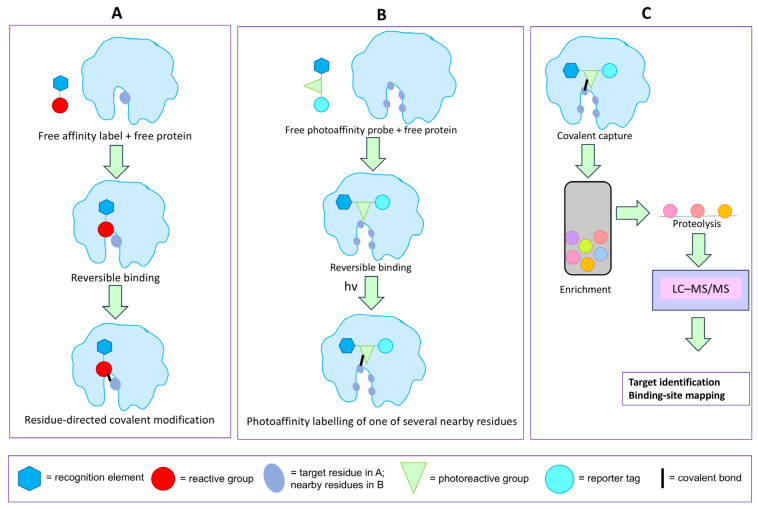
Affinity labelling and photoaffinity labelling as early covalent strategies for target-site identification. (**A**) In affinity labelling, a reactive ligand first binds reversibly to its target and then covalently modifies a selected functional residue in the binding region. (**B**) In photoaffinity labelling, a bound ligand bearing a photoreactive group is activated by irradiation to generate a highly reactive intermediate that crosslinks to nearby residues; therefore, the labelled residue is not necessarily predetermined. (**C**) Subsequent enrichment, proteolysis, and LC–MS/MS analysis enable direct target identification and mapping of ligand-binding regions.

From a chemical biology perspective, during this period, affinity labelling also played an important and early role in identifying ligand binding sites by using reactive ligands that exploit reversible recognition of the target site and then covalently modify functionally important amino acid residues in the same region [59]. Later, this concept was extended by photoaffinity labelling, whereby photoreactive groups were incorporated into bound ligands, so that irradiation generated highly reactive intermediates that covalently trapped adjacent residues, thereby capturing transient ligand–target complexes in cells and membranes [60]. Also, the combination of photoaffinity labelling and mass spectrometric analysis is also recognized as a practical route for direct target identification and structural proteome characterization of bioactive small molecules (Figure 3) [61].

### 2.3. 1980s–1990s (Combinatorial Chemistry, HTS, and Human Genome Project)

**Figure 4:**
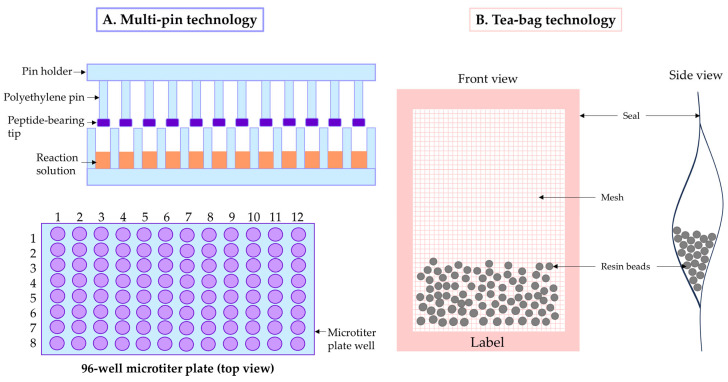
Early parallel solid-phase peptide synthesis methods used in combinatorial chemistry. (**A**) In Geysen’s multi-pin technology, peptides are synthesized in parallel on derivatized polyethylene pins that are immersed into defined wells of a 96-well microtiter plate containing reaction solutions. (**B**) In Houghten’s tea-bag technology, resin is enclosed in labelled porous bags, allowing parallel synthesis through shared processing steps and separate coupling steps.

Combinatorial chemistry is the production of a chemical library including compounds with diverse structures, through systematic, iterative, and covalent linking of different building blocks. In the mid-1980s, combinatorial chemistry was developed by Geysen’s multi-pin technology and Houghten’s tea-bag technology, synthesizing hundreds to thousands of peptides on solid support in parallel (Figure 4) [62,63,64]. Additionally, high-throughput screening (HTS) emerged as a process that uses automation and miniaturized plate assays to test large chemical libraries against biological targets, with a priority on increasing throughput and creating a reliable source of starting material throughout pharma/biotech companies, and is now being used for basic and applied research in academia [65,66]. For example, high-content screening of 57,000 small molecules against intracellular *Mycobacterium tuberculosis* identified dinitrobenzamide derivatives with antimycobacterial activity and linked their activity to inhibition of the decaprenylphosphoryl-β-d-ribose 2′-epimerase subunits 1 and 2 (DprE1/DprE2), required for decaprenylphosphoryl-β-d-arabinose biosynthesis [67]. DprE1 was later established as the active-site cysteine-containing target of benzothiazinones, as discussed later in Section 3.1.6 [68,69].

Meanwhile, the Human Genome Project began and many various pathogen genomes were sequenced, delivering thousands of biological targets with limited chemical starting points for antimicrobial drug discovery [70]. The connection between genome-guided target identification and covalent inhibitor design is discussed in more detail in Section 2.4.

Another important development in this period was the further maturation of bioconjugation chemistry, referring to the covalent derivatization of biomolecules such as proteins, DNA, RNA, and carbohydrates [71]. With continuous advances in chemoselective modification, a significant expansion occurred in its scope and practical utility, especially for the installation of probes, tags, drugs, and other functional moieties onto biological scaffolds [71]. Conceptually, in both bioconjugation and covalent inhibition, a reactive group placed in a suitable position forms a covalent bond with a complementary site on a biomolecule [20,71].

In bioconjugation, new functionalities can be added to a biomolecule by forming a covalent bond, while in covalent inhibition, an electrophilic warhead is positioned to react with a nucleophilic residue in or near the target binding site [20,71]. Fluorescent benzothiazinone analogues provide a preclinical example in which inhibitor-derived probes for DprE1 labelling are generated by attaching a fluorophore to the benzothiazinone scaffold. These probes selectively labelled DprE1 in mycobacteria and actinobacteria, and mutation of the active-site cysteine abolished probe interaction, supporting covalent target specificity [72]. In parallel, some covalent antimicrobials were discovered in this period, such as imipenem (1985) [73], aztreonam (1986) [73], sulbactam (1986) [6], tazobactam (1993) [6], and meropenem (1996) [73] among antibacterials, and artemisinin derivatives widely deployed in the 1990s (e.g., artesunate, artemether) among antiparasitics [74].

### 2.4. 2000s (Genomics and Resistance Concerns)

In the early 2000s, with the expanded availability of microbial genome sequences, organism-specific essential genes have been systematically defined, and the conversion of genomic information into prioritized target lists for antimicrobial drug discovery has become available [75,76]. Filtering gene products to find those genes that are required for growth but are absent in the host was enabled by high-density mutagenesis and hybridization-based approaches, which led to improvements in prioritizing druggable bacterial pathways and reducing the likelihood of host off-target effects [75,76]. A high-density transposon mutagenesis technique was applied to the *Haemophilus influenzae* genome to identify genes required for growth [75]. Later, genes required for optimal growth in *Mycobacterium tuberculosis* were defined by transposon site hybridization (TraSH), and it was revealed that essential genes can be organism-specific [76]. These studies generated validated and prioritized genetic target sets, which supported target-based antimicrobial screening. Some essential pathogen targets contain reactive residues, cofactors, or activation pathways that can provide opportunities for covalent inhibitor design. Examples discussed below include UDP-*N*-acetylglucosamine enolpyruvyl transferase (MurA; target of fosfomycin) [77], DprE1(target of benzothiazones such as BTZ043 and PBTZ169) [68,69,78], enoyl-acyl carrier protein reductase (InhA; target of the isoniazid-derived INH-NAD adduct) [79,80], and viral proteases such as hepatitis C virus (HCV) NS3/4A serine protease(target of boceprevir and telaprevir) [16] and SARS-CoV-2 3C-like protease (3CLpro; target of nirmatrelvir) [18]. 

During this time, treating infections in hospitals became much more difficult because Gram-negative bacteria such as *Klebsiella pneumoniae*, *Escherichia coli*, *Enterobacter* species, and *Pseudomonas aeruginosa* increasingly acquired carbapenemases (β-lactamases capable of hydrolyzing carbapenem antibiotics) [6,81], including KPC enzymes (class A serine carbapenemases) [82], OXA-48–type enzymes (class D oxacillinase carbapenemases, which were first identified in 2001) [83], and NDM-1 (a zinc-dependent metallo-β-lactamase, which was first recognized in 2008) [84]. Carbapenems (e.g., imipenem, meropenem, ertapenem) are broad-spectrum and stable β-lactam antibiotics to many β-lactamases [81].

Subsequently, non-β-lactam inhibitors became greatly important. For this purpose, first, new non-β-lactam agents were designed and developed to bypass β-lactamases entirely. Examples of this period are the nitroimidazoles, pretomanid, and delamanid in tuberculosis; the benzothiazinone BTZ043 against DprE1, or secondly, a β-lactam antibiotic was paired with a non-β-lactam inhibitor that binds to the carbapenemase to keep the antibiotic active [85]. Examples in development at this time included the diazabicyclooctanes (e.g., avibactam/NXL104) and cyclic boronates (precursors to vaborbactam/RPX7009). These inhibitors form reversible and tunable covalent adducts with the serine active site of the enzyme [85]. Other non-β-lactam covalent antimicrobials with different applications to mention in this decade are the HCV protease inhibitors, telaprevir and boceprevir, as antivirals [17,86,87].

**Figure 5:**
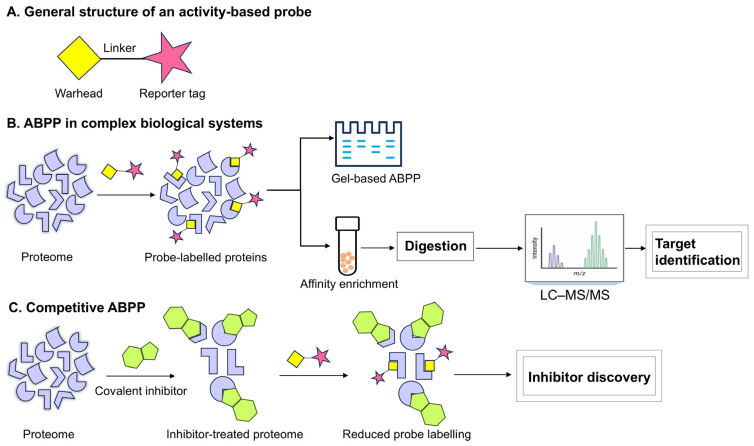
Schematic overview of activity-based protein profiling (ABPP). (**A**) A structure of an activity-based probe, consisting of a warhead, linker, and reporter tag. The warhead reacts with the active-site residue in target proteins, and the reporter tag enables detection or enrichment. (**B**) In complex biological systems, the proteins in the proteome will be activated by activity-based probe labels to generate probe-labelled proteins. These labelled proteins are then analyzed by using gel-based ABPP or by affinity enrichment followed by digestion and LC–MS/MS, allowing target identification. (**C**) In competitive ABPP, first a covalent inhibitor treats the proteome, and if the inhibitor occupies the target protein, subsequent probe labelling is reduced. This reduced probe labelling will be used to evaluate target engagement and support inhibitor discovery.

**Figure 6:**
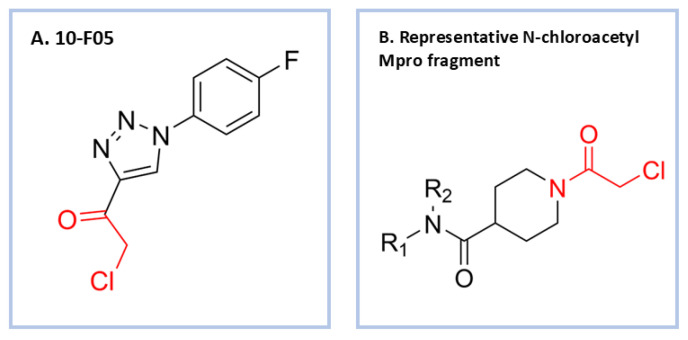
Representative electrophilic fragments from antimicrobial-relevant ABPP/FBDD studies. (**A**) 10-F05, a chloromethyl ketone-containing covalent hit fragment identified by phenotypic screening and competitive ABPP, with FabH and MiaA identified as targets in *Shigella flexneri*. (**B**) Representative N-chloroacetyl-containing electrophilic fragment scaffold identified through crystallographic and electrophilic fragment screening against SARS-CoV-2 Mpro/3CLpro. Electrophilic warheads are highlighted in red.

The discovery and validation of covalent mechanisms in living systems have been accomplished by a set of approaches. Bioorthogonal reactions progressed from the Staudinger ligation (2000) to azide–alkyne “click” chemistry (2002) and then copper-free strain-promoted cycloadditions (2004) and tetrazine ligations (2008) [88,89,90,91,92]. These allow labelling and reading in cells without disrupting native biology. Chemoproteomics, particularly activity-based protein profiling (ABPP), appeared as an affinity-based chromatography approach for identifying small molecules at the proteome level, as a way to confirm the intended target and detect selectivity in many proteins (Figure 5) [93,94,95]. Meanwhile, fragment-based discovery introduced small and electrophilic starting points that could be grown into selective covalent series [96,97]. Weak, reversible binding elements are combined with well-positioned electrophiles by covalent fragment and “tethering” approaches, to capture low-affinity interactions with cysteine or other nucleophilic residues, which can be optimized into potent and selective covalent inhibitors [98]. As research-stage antimicrobial examples, phenotypic screening of cysteine-reactive fragments in combination with competitive ABPP has been used to map and functionally characterize antibacterial hit targets in *Shigella flexneri*, leading to the identification of β-ketoacyl-acyl carrier protein synthase III (FabH) and MiaA tRNA prenyltransferase as targets of the chloromethyl ketone-containing covalent hit fragment 10-F05 (Figure 6) [99]. In another example, crystallographic and electrophilic fragment screening against the SARS-CoV-2 main protease (Mpro/3CLpro) identified covalent active-site fragments, including *N*-chloroacetyl-containing electrophiles as starting points for structure-guided antiviral inhibitor development (Figure 6) [100]. Additionally, residue modification and quantitative analysis of covalent bond formation were identified by X-ray crystal structures and LC–MS/MS [101].

### 2.5. 2010–Present (Renaissance of Covalent Inhibition)

**Figure 7:**
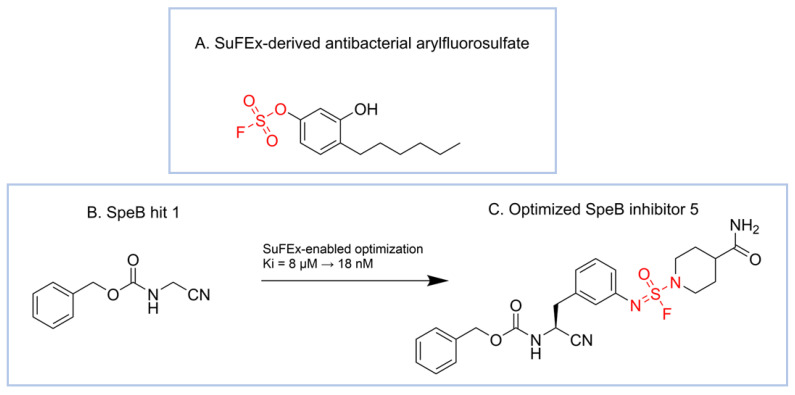
Representative SuFEx antimicrobial examples. (**A**) SuFEx-derived antibacterial arylfluorosulfate reported to show activity against multidrug-resistant Gram-positive bacteria. (**B**) Benzyl (cyanomethyl) carbamate SpeB hit 1. (**C**) Optimized SpeB inhibitor 5 generated through SuFEx-enabled medicinal chemistry, with improved potency from *K_i_* = 8 μM to *K_i_* = 18 nM. SuFEx-related sulfur(VI) groups are highlighted in red.

Since 2010, there has been significant progress in medicinal chemistry as warheads have become more diverse to form more tunable and selective covalent bonds [98,102]. New click-type electrophiles, such as sulfur(VI) fluoride exchange (SuFEx), have expanded selective targeting [90]. Research-stage antimicrobial examples include SuFEx-derived arylfluorosulfates, which have demonstrated antibacterial activity against multidrug-resistant Gram-positive bacteria, including methicillin-resistant *Staphylococcus aureus* (MRSA), vancomycin-resistant *Staphylococcus aureus* (VRSA), and vancomycin-resistant *Enterococcus* (VRE). These arylfluorosulfates selectively killed Gram-positive bacterial strains and showed rapid bactericidal activity, with activity reported both in vitro and in vivo (Figure 7) [103]. Also, SuFEx-enabled high-throughput medicinal chemistry has been applied to SpeB, a cysteine protease virulence factor secreted by *Streptococcus pyogenes*, where a benzyl (cyanomethyl) carbamate hit 1 was optimized to give SpeB inhibitor 5 through installation of a SuFEx-derived iminosulfur oxydifluoride motif, improving inhibitor potency from *K_i_* = 8 μM to *K_i_* = 18 nM and showing activity in a bacteria–host coculture model. (Figure 7) [104].

**Figure 8:**
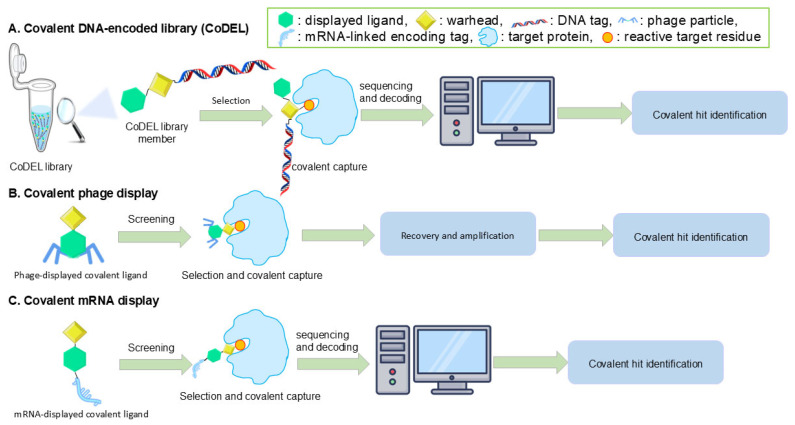
Encoded platforms for covalent hit discovery. Panels (**A**–**C**) show covalent DNA-encoded library (CoDEL), covalent phage display, and covalent mRNA display, respectively. In each case, the displayed ligand contains a warhead and is evaluated against a target protein containing a reactive target residue, with encoding information provided by the DNA tag, phage particle, or mRNA-linked encoding tag.

**Figure 9:**
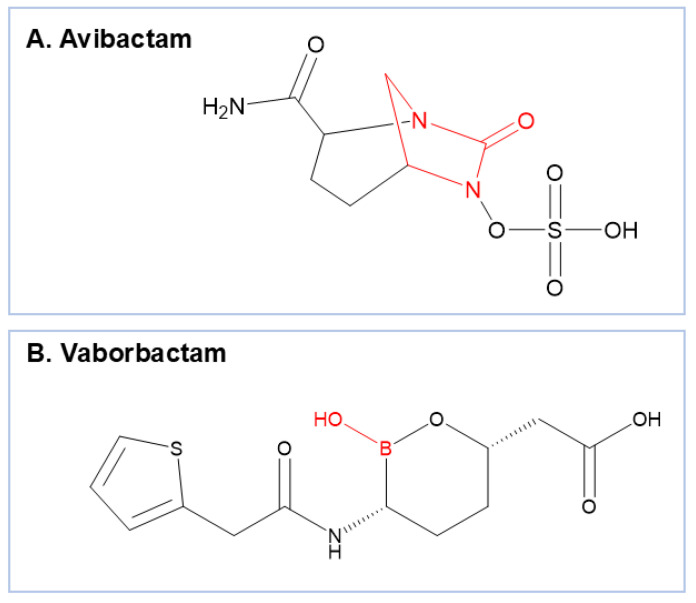
Representative non-β-lactam β-lactamase inhibitors clinically introduced in the modern covalent-inhibition era. (**A**) Avibactam, used in ceftazidime–avibactam. (**B**) Vaborbactam, used in meropenem–vaborbactam. Relevant covalent binding groups are highlighted in red.

During this same period, instead of guessing a target, scientists began using chemoproteomic mapping to chart amino acid residues in natural proteomes that small molecules could engage, shifting target selection to data-driven ligandability at the proteome scale [97,105]. First, the systematic identification of functional cysteines in proteomes was enabled by quantitative cysteine-reactivity profiling. And later, it directly expanded the set of ligandable sites that could be screened in native biological systems [97,105,106,107]. More recently, the expansion of covalent hit discovery by covalent DNA-encoded libraries (CoDELs) and phage/mRNA display platforms with electrophilic warheads have enabled efficient screening against shallow or extended binding sites that are difficult to access with classical small molecule libraries (Figure 8) [98]. In this time, two non-β-lactam reversible β-lactamase inhibitors, ceftazidime–avibactam (2015) and meropenem–vaborbactam (2017), have been clinically validated against resistance enzymes in real patients (Figure 9) [108,109]. Also, tavaborole (2014) was introduced as an antifungal drug by oxaborole tRNA-trapping of leucyl-tRNA synthetase (LeuRS) [12]. A breakthrough in emerging pathogens, nirmatrelvir (part of Paxlovid), an antiviral inhibitor, was able to make a strong and selective reversible covalent bond with the catalytic cysteine of the SARS-CoV-2 3CLpro using a nitrile [18].

As the scientific community has increased its understanding of biological systems, there has been a significant increase in computational power and methods that assist in drug development. Some examples include Fitted [110], which was successful in selecting an appropriate warhead to target a nucleophilic catalytic residue of prolyl oligopeptidase (POP) [111], DUckCov, a virtual screening protocol for covalent binders, and CovalentInDB 2.0, a database for structure- and ligand-based covalent inhibitor designs [112]. Artificial intelligence has also begun to support drug discovery through structure prediction and hit-identification workflows [113], including deep-learning approaches specific for discovery and development of new covalent drugs such as CarsiDock-Cov [114]. For example, AlphaFold2 can predict three-dimensional protein structures from amino acid sequences [115], while AlphaFold3 extends this approach to biomolecular complexes containing proteins, nucleic acids, small molecules, ions, and modified residues [116]. In antimicrobial discovery, deep-learning models have identified antibacterial candidates such as halicin, which was selected from the Drug Repurposing Hub [117], and abaucin, a machine-learning-identified lead with targeted activity against *Acinetobacter baumannii* [118]. Although, direct application of AI or AlphaFold-guided modelling has led to the discovery of new covalent ligands [119], this approach has not yet been applied to covalent antimicrobial inhibitor discovery which remains limited and represents a future opportunity.

Furthermore, covalent targeting of bacterial proteases has been explored as an antimicrobial strategy. In particular, bacterial protease systems such as ClpP have been considered attractive targets for covalent inhibitors, including β-lactone chemotypes. The increased interest in protease targeting for covalent pharmacology has also been driven by natural products such as the syringolins, which were first reported in 1998 and were later shown in 2008 to inhibit the proteasome through covalent modification of the catalytic threonine residue [120,121,122].

## 3. Classes of Non-β-Lactam Covalent Antimicrobials

Below, pathogen classes are categorized based on antibacterial, antifungal, antiparasitic, and antiviral applications, while considering biological and pharmacological limitations that complicate drug discovery across different pathogen types. Bacteria are prokaryotic organisms and therefore contain many cellular structures and pathways that are absent from, or sufficiently distinct from, human cells, allowing more selective pathogen targeting. In contrast, fungal pathogens are eukaryotic and share many cellular features with human hosts, limiting selective target identification and increasing the risk of toxicity [123]. In parasites, target prioritization is further complicated by eukaryotic similarity to the host, life-cycle-stage dependence, target essentiality, and, for obligate intracellular parasites, the need for host cell infection models rather than parasite-only growth assays [124]. In antiviral discovery, candidate targets may include viral proteins or host factors required for viral replication; however, because viruses require host cell machinery to replicate, antiviral assays must be performed in host cell systems and must distinguish direct antiviral effects from host cell toxicity [125]. Representative non-β-lactam covalent antimicrobial agents from these categories are summarized in Table 1.

### 3.1. Antibacterials

#### 3.1.1. Nitrofuran (Nitrofurantoin)

Nitrofurantoin is a nitrofuran prodrug used primarily for uncomplicated lower-urinary-tract infections and it is active against a wide range of Gram-negative and Gram-positive bacteria, including *E. coli*, most species of *Staphylococcus* and *Enterococcus* [126,127]. Genetic determinants of nitrofurantoin resistance in *Enterobacteriaceae* include loss-of-function mutations in the chromosomal genes *nfsA*, *nfsB* (which encode the oxygen-insensitive nitroreductase enzymes NfsA and NfsB, respectively) and *ribE* (encoding 6,7-dimethyl-8-ribityllumazine synthase in riboflavin biosynthesis, which plays a role in reducing the expression of the effect of nitrofurantoin) and the acquired gene complex *oqxAB* (which encodes a multidrug efflux pump OqxAB) [126,127]. Nitrofurantoin toxicity has been associated with rare but potentially serious pulmonary toxicity, which can occur in acute or chronic forms [146].

#### 3.1.2. Isonicotinic Acid Hydrazide (Example: Isoniazid)

Isoniazid, also known as isonicotinic acid hydrazide, is one of the most important first-line drugs used in the treatment of tuberculosis, mainly due to its high potency and selectivity against *Mycobacterium tuberculosis* [79,147]. It is oxidatively activated by KatG, a mycobacterial enzyme [79,148]. Isonicotinyl, which is a radical derived from KatG, forms an INH–NAD adduct after coupling with NAD^+^, which binds to the NADH site of the enzyme InhA (enoyl-ACP reductase, fatty acid synthase-II (FAS-II), an enzyme that plays a critical role in the mycolic acid biosynthesis pathway) as a slow, tight-binding competitive inhibitor and inhibits cell wall synthesis, thereby leading to cell death [79,80,148]. Factors leading to resistance in this pathway include mostly loss or disruption of KatG (e.g., S315T), leading to reduced prodrug activation, changes in InhA (e.g., S94A) or promoter mutations in *inhA* (e.g., the *fabG1*–*inhA promoter variant* −15C → T), reducing the efficacy of the adduct combination [79,148,149]. Additional modifiers (e.g., *ndh* encoding NADH dehydrogenase II, upregulation of the *ahpC promoter*) can alter susceptibility [149].

#### 3.1.3. Cycloserine

Cycloserine is a known antibacterial for the treatment of tuberculosis (TB) and inhibits the biosynthesis of bacterial cell wall peptidoglycan in *M. tuberculosis* [129,130]. A stable d-cycloserine–pyridoxal-5′-phosphate (DCS-PLP) adduct inactivates Alanine racemase (Alr) [129]. DCS also inhibits the enzyme d-Ala-d-Ala ligase (Ddl) through competitive and reversible binding to one of the d-Ala5 binding sites [40]. Typically, resistance to d-cycloserine involves mutations in *alr* and *ddl*, and in some organisms, reduced uptake through the CycA transporter decreases intracellular accumulation of the drug [40,129,130].

#### 3.1.4. Epoxide Phosphonates (Example: Fosfomycin)

Fosfomycin is an epoxy-phosphonate natural product with significant antibacterial activity for treating urinary tract infections (UTIs) [77,131]. It irreversibly inhibits UDP-GlcNAc enolpyruvyl transferase (MurA) [77,150]. MurA catalyzes the first critical step in bacterial peptidoglycan biosynthesis and competes with the substrate phosphoenolpyruvate (PEP) to bind to the active site of MurA [77,150]. Resistance results from *murA* mutations that replace the reactive cysteine or from the absence or downregulation of glycerol-3-phosphate transporter (*glpT*) or hexose-6-phosphate: phosphate antiporter (*uhpT*) transporters that restrict drug entry, as fosfomycin requires active transport into bacterial cells via GlpT and UhpT transporters [131].

#### 3.1.5. Nitroimidazoles (Examples: Delamanid, Pretomanid)

Delamanid and pretomanid are nitroimidazole prodrugs used against *M. tuberculosis*, and the activation process is bioreductive [134]. Their main mode of action is to inhibit the synthesis of mycolic acids, considered a key component of lipids for the formation of the cell envelope of mycobacteria, and also to disturb redox/respiratory balance [134,135]. Delamanid blocks the production of methoxy and ketomycolates, and pretomanid inhibits ketomycolate formation and kills by releasing reactive nitrogen species under hypoxia [134,135]. Its resistance mostly appears due to the mutations in *ddn* or in F_420_-biosynthesis genes (*fbiA*/*B*/*C*/*D*), which impair prodrug activation [135].

#### 3.1.6. Benzothiazinones (Examples: BTZ043, PBTZ169)

Among benzothiazinones (BTZ), BTZ043 has been described as a major inhibitor of DprE1 in *M. tuberculosis* in the presence of FADH2 [68,69,78]. DprE1 is essential for producing decaprenylphosphoryl-d-arabinose (DPA) for arabinan assembly in the cell-wall arabinogalactan [68,69,78]. Substitutions in Cys387 (Cys → Ser/Gly) or active-site nearby residues that hinder adduct formation, and also the redox context alterations that diminish nitroreduction, are resistant to BTZ043 [68,69,78,151]. The second-generation BTZ is PBTZ169 (macozinone), which, despite its improved potency and pharmacokinetics, maintains the same covalent inhibition process and resistance pattern as BTZ043 [78,133,151,152].

### 3.2. Antifungals

#### Oxaborole (Example: Tavaborole)

Tavaborole (5-fluoro-1,3-dihydro-1-hydroxy-2,1 benzoxaborole) is a topical antifungal for the treatment of onychomycosis that inhibits fungal protein synthesis by hindering cytoplasmic leucyl-tRNA synthetase (LeuRS), an aminoacyl-tRNA synthetase, via an oxaborole-tRNA trapping mechanism [86]. The enzyme-bound tRNA is trapped at the editing site and prevents catalytic turnover, thereby inhibiting the synthesis of leucyl-tRNA and subsequently blocking protein synthesis [86]. Resistance to tavaborole occurs due to mutations in the leucyl-tRNA synthetase gene, specifically in the connective polypeptide 1 (CP1)/editing domain that result in impaired trapping of tRNA by the oxaborole at the editing site [141].

### 3.3. Antiparasitics

#### 3.3.1. Difluoromethylornithine (Example: Eflornithine/DFMO)

Difluoromethylornithines (eflornithine/DFMO) are fluorinated ornithine analogues that act as irreversible suicide inhibitors of ornithine decarboxylase (ODC), the first polyamine biosynthesis enzyme in *Trypanosoma brucei* [136,153]. Polyamines are polycationic aliphatic amines that have many functions, but most importantly, are essential for all eukaryotic and prokaryotic cells [136,154]. The predominant resistance that can be noted in *T. brucei* is the loss/reduction in expression of the eflornithine amino-acid transporter in the parasite’s plasma membrane (TbAAT6) [138].

#### 3.3.2. Endoperoxides (Examples: Artemisinin, Artesunate, Artemether)

Endoperoxides contain a 1,2,4-trioxane endoperoxide warhead, and Artemisinin derivatives are antimalarial drugs with a rapid and broad activity against *Plasmodium falciparum* (and activity against blood stages of *P. vivax*) [56]. Since they have a very short half-life, they can be used in artemisinin-based combination therapies (ACTs) with a longer-acting partner (e.g., lumefantrine, mefloquine, piperaquine) to ensure cure and suppress relapse [56]. The dominant cause of partial resistance to endoperoxides is Kelch13 propeller mutations in *P. falciparum*; see Section 5.4 for mechanisms [87].

#### 3.3.3. Nitroimidazoles (Examples: Metronidazole, Tinidazole)

Metronidazole is used to treat anaerobic protozoal infections associated with inflammatory disorders of the gastrointestinal tract, including giardiasis and amebiasis [15,139,140]. In general, protozoal resistance to nitroimidazoles involves impaired bioreductive activation, reduced electron transfer from pyruvate ferredoxin oxidoreductase to ferredoxin, and subsequent decreased activity associated with higher intracellular oxygen and more extensive redox reprogramming that oxidizes nitro radicals and limits the formation of cytotoxic nitroso and hydroxylamine intermediates [14,140].

### 3.4. Antivirals

#### 3.4.1. α-Ketoamides (Examples: Boceprevir, Telaprevir)

Boceprevir and telaprevir are antivirals for the treatment of hepatitis C virus (HCV) genotype 1, inhibiting the viral serine protease NS3/4A [17,155]. They were among the first direct-acting antivirals (DAAs) approved for chronic HCV infection, and their use in combination with pegylated interferon and ribavirin has been shown to significantly improve sustained virologic response rates compared with interferon-based therapy alone, although this comes at the cost of complicated dosing schedules and substantial adverse effects. Resistance to these α-ketoamides results from amino acid substitutions in NS3 that weaken inhibitor interaction by altering the conformation of the binding subsites [17,155,156].

#### 3.4.2. Nitrile (Nirmatrelvir)

Nirmatrelvir, as a reversible covalent inhibitor of the SARS-CoV-2 3C-like protease (3CLpro), blocks the viral polyprotein processing and replication [18]. Nirmatrelvir is administered orally in fixed-dose combination with ritonavir (Paxlovid), which acts as a pharmacokinetic booster via CYP3A4 inhibition, and is approved for the early treatment of mild-to-moderate COVID-19 in adults at a high risk for progression to severe disease [19,157]. Resistance to nirmatrelvir emerges through mutations in the binding site of the virus’s 3CLpro, which is mainly at E166 (often with L50F) and also substitutions in S1/S2/S4 that weaken inhibitor binding [144,145].

Beyond protease-targeted antivirals, nucleos(t)ide reverse transcriptase inhibitors illustrate antiviral activity through incorporation into viral genetic material. This strategy has a long history in antiretroviral therapy, beginning in 1987 with zidovudine (AZT), which was approved by the FDA as the first antiretroviral drug for AIDS treatment [158]. Nucleos(t)ide reverse transcriptase inhibitors require intracellular activation to their active phosphorylated forms and can be incorporated into viral DNA by HIV-1 reverse transcriptase, leading to chain termination [159]. Biktarvy is a clinically used single-tablet HIV-1 combination regimen containing bictegravir, emtricitabine, and tenofovir alafenamide. In this regimen, bictegravir is an integrase strand transfer inhibitor, while emtricitabine and tenofovir alafenamide are nucleos(t)ide reverse transcriptase inhibitors [160]. Unlike warhead-based covalent enzyme inhibitors, these agents act through covalent incorporation into viral DNA rather than covalent modification of a protein target.

## 4. Warhead Mechanisms for Non-β-Lactam Covalent Antimicrobials

**Figure 10:**
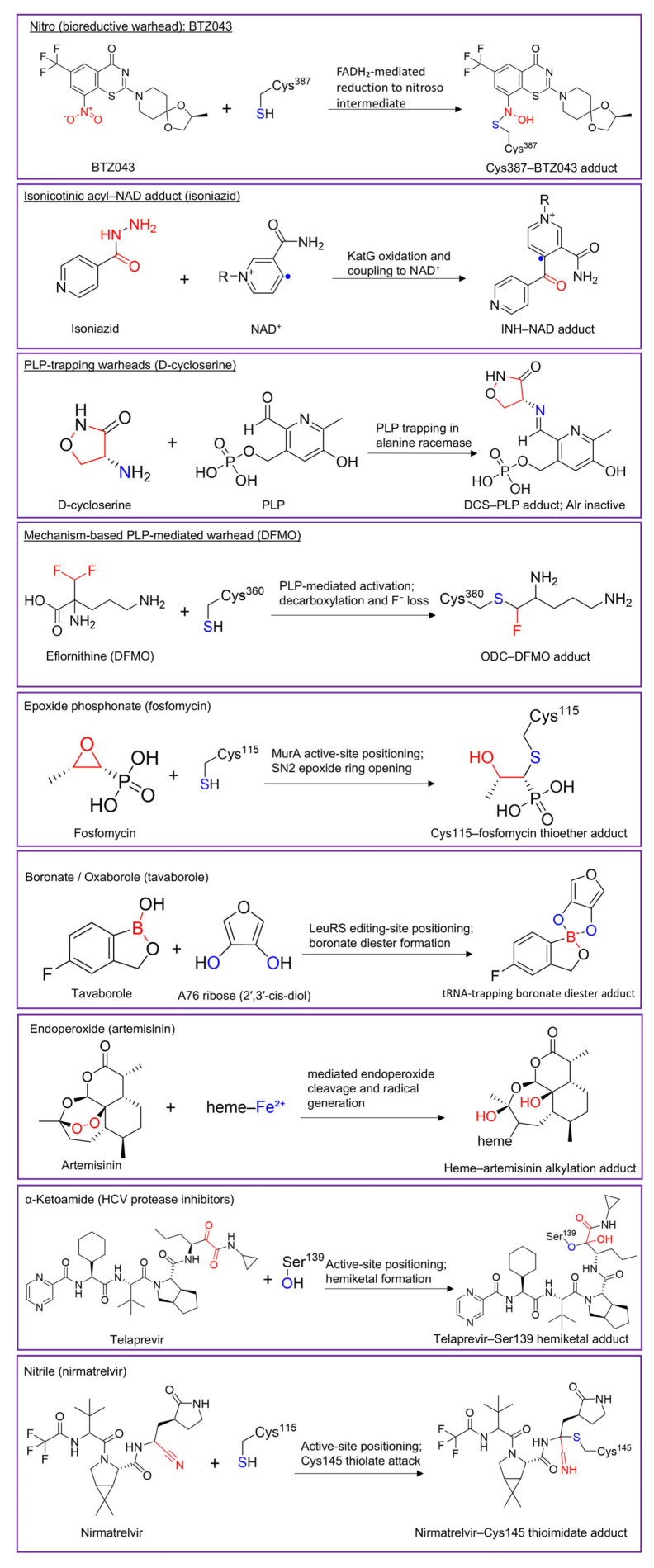
Red indicates the warhead. Blue indicates the directly reacting partner, usually the nucleophile. Exceptions are isoniazid (reactive NAD^+^ site), tavaborole (A76 ribose 2′,3′-cis-diol oxygens), and artemisinin (heme Fe^2+^ activator).

A covalent bond forms between an inhibitor’s warhead (the electrophilic moiety) and a biological nucleophile, typically at a residue in or near an enzyme’s active site [22,23,24]. Historically, targeted covalent inhibitors have mainly relied on cysteine as the nucleophilic handle, but over the past decade, the exploration of a diverse array of warheads has enabled the selective engagement of non-cysteine nucleophiles as well [161]. In covalent inhibition, selectivity and potency are both critical. Selectivity arises from the pre-organized binding pocket and from regulation of residue nucleophilicity via pK_a_, hydrogen-bonding networks, and catalytic motifs [22,23,24]. In parallel, potency reflects how efficiently a properly tuned warhead forms (and, if reversible, maintains) the covalent adduct (Figure 10) [22,23,24].

Covalent binding can be reversible or irreversible. Reversible covalent adducts have occupancy that tracks recognition and residence time (e.g., nitriles or α-ketoamides) [18,142,143]. On the other hand, irreversible adducts do not turn over on biological timescales and therefore demand tighter structural recognition and careful exposure control, often aided by on-site activation (e.g., epoxides, mechanism-based PLP warheads, bioreductive nitro groups) [11,40,77,129]. Here are the key warhead types used by the non-β-lactam covalent antimicrobials described in Section 3.

- Nitro (bioreductive warheads): The nitro group is a bioreductive prodrug warhead in nitrofurans, nitroimidazoles, and benzothiazinones. Intracellular nitroreductases reduce nitro groups to nitro-radical anions, which, under low O_2_ conditions, are further reduced to nitroso/hydroxylamine electrophiles; these species form irreversible adducts with proteins and DNA. Activation: (i) oxygen-insensitive nitroreductases NfsA/NfsB in many bacteria, (ii) deazaflavin F_420_-dependent nitroreductases (e.g., Ddn) in mycobacteria, (iii) ferredoxin-linked nitroreductases in anaerobic protozoa, and (iv) reduction in in-pocket flavin (FADH_2_)-mediated generates in situ nitroso for covalent capture of an active-site Cys (e.g., Cys387) [126,127].
- Isonicotinic acyl–NAD adduct (isoniazid): Isoniazid is activated by the mycobacterial enzyme KatG, a multifunctional catalase-peroxidase, to an isonicotinyl radical that, upon reaction with NAD^+^, forms a stable INH-NAD adduct [79,149]. This adduct binds to the active site of InhA, thereby blocking the FAS-II mycolic-acid biosynthesis pathway [79,149].
- PLP-trapping warheads (D-cycloserine): In peptidoglycan biosynthesis, D-cycloserine targets PLP-dependent enzymes. In alanine racemase, It forms a stable DCS-PLP adduct at the catalytic lysine site that effectively traps the cofactor and leads to enzyme irreversible inactivation [40,129,130]. It also acts as a competitive inhibitor in inhibiting D-Ala ligase at one of the D-Ala binding sites [40,129,130].
- Mechanism-based PLP-mediated warhead (DFMO): Difluoromethylornithine (DFMO) is a suicide inhibitor for PLP-dependent ornithine decarboxylase (ODC) [136,153,154]. DFMO forms an external aldimine with PLP, and the enzyme-catalyzed decarboxylation of this aldimine triggers fluoride loss to generate an in situ electrophile that irreversibly inactivates ODC in a turnover and time-dependent manner. Therefore, the activation is PLP-mediated enzyme turnover [136,137,153,154].
- Epoxide phosphonate (fosfomycin): A strained epoxide carried by fosfomycin is linked to a phosphonate that mimics phosphoenolpyruvate (PEP). In MurA, the S_N_2 attack on the epoxide carbon is mediated by the active-site cysteine (Cys115), which opens the ring and forms an irreversible thioether adduct. Activation is active-site positioning, while exposure can be gated by transporter-mediated uptake [77,131,150].
- Boronate/Oxaborole (tavaborole): Oxaboroles such as tavaborole contain a boron-centred warhead that forms a reversible boronate diester with cis-diols. In LeuRS, a boronate diester with the 2′,3′ cis-diols of the A76 ribose of tRNALeu in the editing pocket forms by tavaborole, trapping the tRNA and blocking aminoacylation [24,86,141]. Activation depends on the active-site geometry/positioning of the tRNA 3′ [24,86,141].
- Endoperoxide (artemisinins): 1,2,4-trioxane endoperoxide is the radical-generating warhead of artemisinin and its derivatives. Alkylation of heme, as well as multiple parasite proteins, occurs via the generation of short-lived carbon-centred radicals after Fe^2+^ cleaves the O-O bond of the endoperoxide [56,87]. The activation depends on the presence of heme derived from hemoglobin digestion [56,87].
- α-Ketoamide (HCV protease inhibitors): The α-ketoamide warhead in HCV NS3/4A protease inhibitors, such as boceprevir and telaprevir, is attacked by the catalytic serine, resulting in the formation of a reversible hemiketal stabilized in the oxyanion hole [17,155,162]. Since the covalent adduct can hydrolyze, occupancy is dictated by both noncovalent fit in the binding subsites and the electronics of the α-ketoamide. Activation occurs without an additional enzyme-dependent step via active-site binding and positioning [17,155,162].
- Nitrile (nirmatrelvir): The nitrile warhead of nirmatrelvir reacts with the catalytic cysteine (Cys145) of the SARS-CoV-2 3C-like protease (3CLpro) [18]. A reversible thioimidate is formed from the attack of the catalytic cysteine on the nitrile carbon with the His-Cys dyad generated form the nucleophilic thiolate, and the activation occurs via active site binding and positioning [18].

Notably, the warheads discussed in this review differ in electrophilic reactivity, reversibility, and residue/cofactor selectivity [22,23,24]. Nitro-containing agents and endoperoxides are activation-dependent systems; nitro warheads rely primarily on enzymatic/redox activation, whereas endoperoxides undergo Fe^2+^/heme-associated radical formation [56,134,135]. Epoxide and nitrile warheads require precise active-site positioning to react selectively with catalytic cysteine residues, as shown by fosfomycin–MurA and nirmatrelvir–3CLpro interactions [18,77]. α-Ketoamides and nitriles illustrate reversible covalent inhibition through adduct formation with catalytic serine and cysteine residues, respectively [17,18,143]. Oxaboroles trap tRNA through boronate ester formation with ribose cis-diols [12]. PLP-dependent examples, including D-cycloserine and DFMO, rely on enzyme-assisted cofactor chemistry rather than direct electrophile exposure [9,137].

## 5. Resistance Mechanisms in Non-β-Lactam Covalent Antimicrobials

Across bacteria, fungi, parasites, and viruses, resistance to non-β-lactam covalent antimicrobials involves a limited set of strategies. Below we summarize these strategies that are related to the agents discussed in Section 3 and Section 4.

### 5.1. Target Modification

At or near the reactive site, point substitutions or local structural changes can misalign or weaken the nucleophile (e.g., serine or cysteine and their supporting residues), or destabilize the covalent transition state or adduct [16,142,163]. In HCV, NS3 substitutions (V36M, T54A/S, R155K/T, A156T/V) reduce the alignment of α-ketoamide inhibitors (boceprevir, telaprevir), thereby weakening hemiketal formation and reducing inhibitor affinity [16,142]. In SARS-CoV-2 3CLpro, substitution at E166 with L50F can lead to reduced susceptibility to nirmatrelvir by altering the geometry of the active site and disrupting the S1, S2, and S4 substrate-binding pocket [144]. In tavaborole, in vitro resistance studies showed that *Trichophyton rubrum* mutants displayed 4- to 8-fold increases in tavaborole MIC. Although the specific mutation sites in *T. rubrum* were not mapped in that study, the authors noted that previous studies in *Saccharomyces cerevisiae* and *Escherichia coli* linked tavaborole resistance to mutations in the leucyl-tRNA synthetase-encoding gene that alter the editing site or affect hydrolytic editing activity, consistent with reduced oxaborole-mediated tRNA trapping [141]. In benzothiazinones, substitution of DprE1 Cys387 to Ser/Gly in *M. tuberculosis* confers resistance to BTZ043 and PBTZ169 by eliminating the formation of nitroso-mediated adducts [68,69]. In fosfomycin, mutations at Cys115 in MurA eliminate epoxide attack and reduce sensitivity [77,131,150]. In D-cycloserine, mutations in alanine racemase (*alr*) and D-Ala–D-Ala ligase (*ddl*) reduce DCS-PLP adduct formation [40,129,130]. In isoniazid, mutations in the InhA (e.g., S94A) or promoter mutations (e.g., *fabG1*–*inhA promoter* −15C → T) reduce binding and effectiveness of the INH–NAD adduct [79,149].

### 5.2. Loss or Bypass of Prodrug/Cofactor Activation

In some cases, covalent bond formation requires an activating enzyme, cofactor or metal; disrupting of this activation step prevents productive adduct formation [163]. In *M. tuberculosis*, nitroimidazoles such as pretomanid and delamanid require bioreductive activation by a Ddn/F_420_ system. Therefore, mutations in *ddn* or in *fbiA*, *fbiB*, *fbiC*, and *fbiD* (F_420_-biosynthesis genes) can reduce drug activation and lower susceptibility [134,135]. For example, the pretomanid-resistant *M. tuberculosis* strain (N0008) carries an S78Y substitution in Ddn and showed a 64-fold increase in pretomanid MIC compared with the reference strain (H37Rv), from 4 to 256 μg/mL, while remaining susceptible to delamanid [135]. In anaerobic protozoa, reduction in *nfsA*/*nfsB* or ferredoxin-linked nitroreductases limits nitro bioreduction [14,140]. In *Plasmodium* spp., changes in heme handling and hemoglobin endocytosis modulate the availability of Fe^2+^–heme needed to cleave the endoperoxide bridge of artemisinins, thereby contributing to partial resistance phenotypes [56,87]. In addition, for isoniazid, KatG loss prevents the formation of the INH-NAD adduct by disrupting the oxidation of the prodrug to the isonicotinyl radical [79,136,149].

### 5.3. Reduced Intracellular Accumulation (Uptake Loss and Efflux Gain)

Reduced intracellular drug levels can lead to resistance, independent of the drug’s intrinsic potency. This typically involves loss, downregulation, or mutation of uptake routes and amplification of efflux systems [163,164]. In *T. brucei*, loss of the amino-acid transporter TbAAT6 lowers cellular uptake of eflornithine (DFMO) [138]. In Enterobacterales (e.g., *E. coli*, *K. pneumoniae*), loss or downregulation of the sugar-phosphate transporters GlpT/UhpT prevents fosfomycin uptake [131]. Efflux can also reduce intracellular exposure to covalent antimicrobials; in uropathogenic *K. pneumoniae*, nitrofurantoin resistance has been associated with the multidrug efflux pumps AcrAB and OqxAB, including isolates with nitrofurantoin MIC values of 128 μg/mL [128]. In mycobacteria, reduced CycA transporter activity can lower D-cycloserine uptake and decrease intracellular exposure, as supported by studies linking a *cycA* point mutation to D-cycloserine resistance in *Mycobacterium bovis* BCG [165].

### 5.4. Metabolic Rewiring, Redox Buffering, and Proteostasis Adaptation

A subset of cells can weaken covalent damage by rebalancing pathway flux, oxygen/redox state, or protein quality control [163,164]. In *P. falciparum*, Kelch13-linked phenotypes are associated with altered hemoglobin endocytosis/metabolism and redox/proteostasis shifts, yielding the clinical picture of partial artemisinin resistance [56,74,87]. In anaerobic protozoa, increased intracellular O_2_ and broader redox reprogramming quench nitro-derived radicals and limit accumulation of cytotoxic nitroso/hydroxylamine intermediates [14,140]. In Enterobacterales, *ribE* perturbations reduce flavin pools and indirectly depress nitrofuran activation [131,132].

### 5.5. Phenotypic Expression and Fitness Costs

Resistance does not always make a drug completely ineffective (partial resistance), it often makes the effect more subtle, where the drug still works, just slower or less [74,164]. In *P. falciparum*, artemisinin endoperoxides resistance manifests as a delay in parasite clearance, so a concomitant drug with ACT becomes critical to complete the parasite killing process [56,74,87]. In contrast, mutations in viral proteases (HCV NS3, SARS-CoV-2 3CLpro) are associated with fitness costs; when drugs are not available, mutated viruses replicate less efficiently, which reduces the persistence of these mutations in the population [16,144,145].

### 5.6. Enzymatic Drug Inactivation

Some pathogens chemically and independently of transport or mutation deactivate covalent warheads before they reach their target. For fosfomycin, FosA/FosA3 metalloenzymes (Mn^2^-dependent glutathione-utilizing epoxide-opening enzymes) generate an inactive conjugate and confer high-level resistance in Gram-negative bacteria [11,132,166].

These resistance mechanisms can create trade-offs between drug survival and microbial fitness. Although resistance mutations may improve survival under drug pressure, they often reduce microbial fitness when the drug is absent; therefore, low-cost or no-cost resistance mutations are more likely to persist in microbial populations [167,168].

The magnitude of this fitness cost depends in part on the genetic basis of resistance; for example, chromosomal resistance mutations generally impose greater fitness costs than plasmid-acquired resistance [168]. This is particularly relevant for covalent antimicrobials because resistance can involve alteration of reactive target residues, reduced prodrug activation, altered redox or cofactor-linked activation pathways, reduced uptake, or increased drug inactivation [68,69,131,134,135].

Mutations that prevent covalent adduct formation at an active-site nucleophile may also compromise normal enzyme function when the same residue or nearby catalytic environment contributes to substrate recognition, product release, catalytic efficiency, or protein stability [68,69,77]. Strongly fitness-impaired resistant populations may adapt more slowly or follow evolutionary routes that first restore growth before further increasing drug resistance [169]. Therefore, resistance to covalent antimicrobials should be viewed not only as reduced drug susceptibility, but also as a balance between avoiding covalent modification and preserving the biological function of the targeted or activating pathway.

## 6. Mitigation Strategies: Design and Clinical

Resistance can reduce the effectiveness of covalent antimicrobials. To this end, we will discuss methods to reduce or completely prevent resistance. These modern mitigation strategies operate on two fronts: design level (how the molecule is built) and clinical level (how it is deployed in patients) [93,170,171]. Below we summarize mitigation strategies related to resistance mechanisms in Section 5.

### 6.1. Design Level

At the design level, resistance mitigation focuses on building covalent inhibitors that retain target engagement while reducing liabilities such as excessive intrinsic reactivity, dependence on a single mutable residue, fragile prodrug activation, poor intracellular accumulation, or enzymatic inactivation. Below, various strategies illustrate how covalent warhead placement, target selection, and scaffold optimization can be tuned to potentially reduce resistance while maintaining antimicrobial activity.

- Chemoproteomics-guided covalent fragments for placement and selectivity: Chemoproteomic profiling of ligandable residues (e.g., proteome-wide cysteine reactivity) is used to choose a “safe” nucleophile and calibrate intrinsic warhead reactivity; then, low-reactivity electrophilic fragments, often bearing tunable or reversible warheads such as nitriles, boronates/oxaboroles, or sulfonyl (sulfur(VI)) fluorides, are grown into inhibitors that maintain strong, selective occupancy while limiting pan-reactivity and raising the genetic barrier to escape [93]. As an example, proteome-wide cysteine mapping coupled with a large covalent-fragment screen on SARS-CoV-2 3CLpro yielded several Cys145-anchored starting points that were rapidly optimized into resistance-aware scaffolds [100].
- Mitigating target-modification resistance: In protease inhibitors, designing a substrate envelope can maintain ligand bulk within the conserved envelope, thereby minimizing contacts with the R155/A156 hot spots in HCV NS3/4A and reducing susceptibility to target-modification resistance [143]. Also, restoring potency against E166V alone or in combination with L50F by incorporating structure-guided “strategic flexibility” in SARS-CoV-2 3CLpro inhibitors while preserving the productive interaction of Cys145 can overcome resistance to nirmatrelvir in preclinical studies [172].
- Bypassing single-residue covalent anchors: When covalent engagement relies on the modifiable residue DprE1 Cys387, C387S/G blocks BTZ adduction; mitigation is to switch to noncovalent DprE1 inhibitors that occupy the FAD-proximal pocket via H-bonds/π-stacking/hydrophobic contacts, thereby decoupling activity from Cys387 [173].
- Fragile activation steps: When the effect of a prodrug is compromised by the loss of a single activator, direct-acting alternatives such as diazaborines (AN12855), which inhibit InhA without KatG/cofactor activation, can bypass the defect in preclinical models [174].
- Intracellular accumulation mitigation: Shape, polarity, and ionization can be engineered to evade broad efflux and favour uptake. For example, to prevent resistance to fosfomycin, including the loss of GlpT/UhpT, fosfomycin can be combined with ceftazidime-avibactam, thereby restoring activity and suppressing the emergence of resistance in multidrug-resistant *P. aeruginosa* [175].
- Mitigating metabolic/redox adaptation: A bacterio-modulation approach blocks the bacterial itaconate degradation pathway (Ict/Ich/Ccl) using pantetheine-linked prodrugs that are converted to CoA analogue inhibitors in bacteria (PanK-I/PPAT/DPCK), thereby resensitizing *Salmonella enterica* to macrophage-derived itaconate without intrinsic antibacterial activity [176].
- Evading enzymatic drug inactivation: In some Gram-negative bacteria, FosA metalloenzymes inactivate the drug by opening the epoxide of fosfomycin. Mitigation proceeds when FosA is directly inhibited with small molecules such as ANY1 or phosphonoformate/foscarnet to restore fosfomycin activity, while the warhead is redesigned through electrostatic shielding or repositioning of the reactive group to reduce accessibility to the enzyme [177]. This approach has also been used for targeting enzymatic inactivation of tetracyclins by tetracyclin destructases (TD). Cyclopropylamines and propargylamines were developed to attack FAD in the binding pocket of TD, which prevented FAD from being recycled into FADH_2_, thus preventing TDs from inactivating tetracyclins. The addition of this deactivating warhead led to a 2–32× increase in tetracyclin activity in the presence of TDs [178].

### 6.2. Clinical Level

At the clinical level, resistance mitigation depends on how covalent antimicrobials are deployed after resistance mechanisms are known or anticipated. These strategies include switching within or outside a target class, using diagnostic information to bypass failed activation pathways, combining agents to restore intracellular exposure or activity, and optimizing dosing regimens to reduce selection of resistant subpopulations.

- In-class switching guided by cross-resistance data: If the activity 3CLpro inhibitor is reduced by the variants, another variant of the same class can be switched that grips different subsites (e.g., switching between nirmatrelvir and ensitrelvir) [172].
- Anchor-loss contingency within the same target (pairs with 6.1.3): In cases where covalent bond formation depends on a single residue, clinical performance is enabled by the availability of noncovalent supports (e.g., quabodepistat/OPC-167832, TBA-7371) [173].
- Diagnostics-guided bypass of failed activation (pairs with 6.1.4): If the single activator or cofactor of a covalent prodrug is compromised, such as the KatG, F_420_ systems, a direct-acting inhibitor that engages the same target can be used to maintain efficacy (e.g., diazaborines/AN12855 for InhA) [174].
- Combination therapy to offset uptake loss (pairs with 6.1.5): When loss of the transporter reduces intracellular drug levels, combination with a synergistic partner restores bactericidal activity, e.g., fosfomycin plus ceftazidime-avibactam in multidrug-resistant (MDR) *P. aeruginosa* [175].
- Host-context adjuvancy in intracellular infections (pairs with 6.1.6): In the bacterial CoA pathway (PanK/PPAT/DPCK), pantetheinamides that are bioactivated disrupt CoA balance by engaging the PanD-PanZ complex (which is absent in host cells), thereby selectively suppressing intracellular *Salmonella* and reducing organ burden in a mouse colitis model [179].
- Countermeasures to enzymatic inactivation (pairs with 6.1.7): When a covalent agent is weakened by inactivating enzymes (e.g., FosA vs. fosfomycin), a direct enzyme inhibitor with exposure optimization can be used to restore the covalent effect [177].
- Pharmacokinetic and pharmacodynamic regimen design: In some cases, it is possible to adjust doses and times to set drug levels above the mutation-preventing concentration, thereby minimizing the time in the mutation selection window, which leads to a reduction in resistant mutations in the first step [170].
- Collateral-sensitivity sequencing: After enrichment of LeuRS editing site mutants by tavaborole, those mutants become selectively vulnerable to norvaline; tavaborole → norvaline sequencing is used to suppress the resistant subpopulation [171].

## 7. Conclusions and Future Perspectives

Covalent inhibition is the formation of a bond between an electrophilic warhead and a nucleophilic residue at or near a target’s active site. Potency and selectivity arise from coupling shape-driven recognition with reaction chemistry. This review addresses key historical inflexion points that redirected attention beyond β-lactams scaffolds. These key points include the move from phenotypic screens to rational design under more stringent safety norms, pharmacophore and affinity/photoaffinity tools (Figure 3); combinatorial chemistry and HTS; genome-guided target setting in the midst of the β-lactamase crisis; and, more recently, chemoproteomics, click/SuFEx and fragment-guided covalency, and AI-aided modelling [115,119,180,181]. We then reviewed non-β-lactam covalent drugs across antibacterial, antifungal, antiparasitic, and antiviral applications, placing them on a historical timeline (Figure 1), and organizing their classifications, warheads, primary targets, covalency (reversible vs. irreversible), activation requirements, key resistance routes, and the relevant mitigation strategies at both design and clinical levels. Looking forward, covalent inhibitor design could lead to advances in covalent drug discovery with the continued expansion of tunable warheads, improved proteome-wide ligandability mapping, broader targeting of residues beyond cysteine, and tighter integration of chemoproteomic and computational workflows [161,182,183] Selective covalent reactivity is not an isolated phenomenon, but rather a recurring strategy in nature. Throughout the historical development outlined here, natural products revealed distinct covalent mechanisms and provided inspiration for the discovery of new warheads, new target classes, and more resistance-aware covalent scaffolds. In this regard, natural products with covalent modes of action should continue to be important sources of inspiration for future directions [161].

Inspiration for new covalent antibiotics can also be taken from currently known scaffolds with the incorporation of warheads. A recently developed database for such antibiotics is AntibioticDB, wherein one can search for antibiotics in different stages of clinical development and which is publicly accessible [184].

## Figures and Tables

**Table 1:** Non-β-lactam covalent antimicrobial agents organized by class, status, pathogen type, warhead, target, covalency, activation requirement, and resistance routes. Status: M = marketed/clinically approved; C = clinical stage/in clinical trials; W/D = withdrawn/discontinued. Relevant covalent warheads are highlighted in red. Symbols: → indicates conversion or progression to the indicated product or effect; ↑ indicates an increase or upregulation; ↓ indicates a decrease or downregulation.

Class	Status	Pathogen Type	Warhead	Primary Target	Covalency	Activation	Key Resistance Routes	Key References
**Nitrofuran** Example: Nitrofurantoin (structure shown) 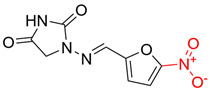	M	Bacteria (Urinary tract infection)	Nitro (bioreductive) → radicals/nitroso; DNA/RNA/protein adducts	Multiple proteins (ribosomal/enzymatic)	Irreversible	Bacterial nitroreductases (NfsA/NfsB)	*nfsA*/*nfsB* loss; *oqxAB* efflux; *ribE*/redox limitation	[126,127,128]
**Isonicotinyl Hydrazides** Example: Isoniazid (structure shown) 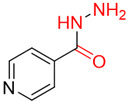	M	Bacteria (Tuberculosis)	Hydrazide → INH–NAD covalent adduct	InhA (enoyl-ACP reductase; FAS-II)	Reversible (slow-binding)	KatG oxidation couples INH to NAD^+^	*katG* loss/S315T; *inhA* S94A; *fabG1*–*inhA promoter* −15C → T; ahpC ↑	[10,79,80]
**Cycloserine** Example: d-Cycloserine 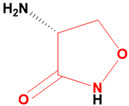	M	Bacteria (Tuberculosis and others)	Oxazolidin-2-one (d-Ala mimic) → PLP-trapping adduct	Alanine racemase; (Alr; PLP); d-Ala–d-Ala ligase (Ddl; ATP)	Irreversible for Alr, reversible for Ddl	Enzyme-assisted via PLP chemistry	*alr*/*ddl* mutations or overexpression; ↓ uptake (*cycA*)	[9,40,129,130]
**Epoxide Phosphonates** Example: Fosfomycin (structure shown) 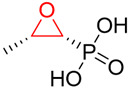	M	Bacteria (Urinary tract infection and others)	Epoxide (S_N_2 ring opening)	MurA (active-site Cys; *E. coli* Cys115)	Irreversible	Intrinsic electrophile; transport-dependent exposure (GlpT/UhpT)	*murA* substitution; *glpT*/*uhpT* loss/downregulation	[131,132]
**Benzothiazinones** Examples: BTZ043 (structure shown), PBTZ169 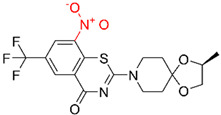	C	Mycobacteria	Nitro → nitroso; Cys adduct	DprE1 (e.g., Cys387 in *M. tuberculosis*)	Irreversible	DprE1-bound FADH_2_ (in-active-site nitroreduction)	*dprE1* Cys387 → Ser/Gly; that hinder nitroreduction/adduct formation; reduced DprE1-mediated nitroreduction	[68,69,78,133]
**Nitroimidazoles** Examples: Delamanid, Pretomanid (structure shown)** 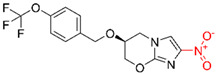 **	M	Mycobacteria	Nitro → radicals/nitroso; adduct lesions	Mycolic-acid synthesis; redox/respiration under hypoxia	Irreversible	Deazaflavin (F_420_)-dependent Ddn nitroreduction (*ddn*, *fgd1*, *fbiA*/*B*/*C*/*D)*	*ddn* loss/mutation; *fgd1* and *fbiA*/*B*/*C*/*D* defects; impaired F420 biosynthesis/function.	[134,135]
**Difluoromethylornithine** Example: Eflornithine (structure shown) 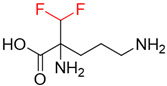	M	Parasite (*Trypanosoma brucei*)	Mechanism-based (enzyme-catalyzed) adduct	Ornithine decarboxylase (PLP-dependent)	Irreversible	Requires enzyme turnover	TbAAT6 transporter loss/reduced uptake	[13,136,137,138]
**Nitroimidazoles** Examples: Metronidazole (structure shown), Tinidazole 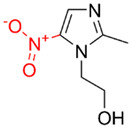	M	Protozoa/anaerobes	Nitro (bioreductive) → radicals/nitroso; DNA/protein adducts	Multiple (DNA, redox enzymes)	Irreversible	Nitroreductase activation (pyruvate:ferredoxin oxidoreductase (PFOR) → ferredoxin) under low O_2_	↓ nitroreductase/e^−^ donors; ↑ O_2_ tension; oxidative-stress adaptation	[14,139,140]
**Endoperoxides** Examples: Artemisinin (structure shown), Artesunate, Artemether 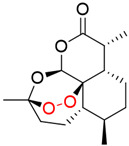	M	Parasite (*Plasmodium* spp.)	Endoperoxide → Fe^2+^-triggered radicals	Heme/Fe^2+^-heme complexes; multi-protein alkylation	Irreversible	Fe^2+^-mediated endoperoxide cleavage	kelch13 variants; altered redox/hemoglobin metabolism	[56,74,87]
**Oxaborole** Example: Tavaborole (structure shown) 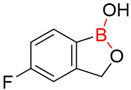	M	Fungi (onychomycosis)	Boronic acid/boronate (tRNA-trapping)	LeuRS CP1/editing site via tRNALeu A76 adduct	Reversible	Active-site geometry/positioning	LeuRS mutations	[12,141]
**α-Ketoamides** Examples: Boceprevir, Telaprevir (structure shown) 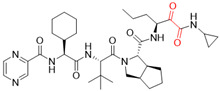	W/D	Virus (Hepatitis C, GT1)	α-Ketoamide (amide C=O–α–ketone C=O; Ser-hemiketal)	HCV NS3/4A serine protease	Reversible	Active-site positioning/fit (no enzyme-gated chemistry)	NS3 substitutions: R155K/T, A156T/V, V36M, T54A/S	[16,17,142,143]
**Nitrile** Example: Nirmatrelvir (structure shown) 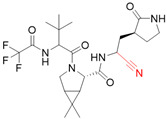	M	Virus (SARS-CoV-2)	Nitrile—thioimidate with Cys	SARS-CoV-2 3CLpro (Cys145)	Reversible	Active-site positioning/fit	Protease mutations	[18,144,145]

## Data Availability

No new data were created or analyzed in this study.
